# Socially responsible investing: from the ethical origins to the sustainable development framework of the European Union

**DOI:** 10.1007/s10668-021-01375-3

**Published:** 2021-04-07

**Authors:** Alice Martini

**Affiliations:** Dipartimento Di Economia E Management, Università Di Pisa, via Ridolfi, 10, 56124 Pisa, Italy

**Keywords:** Socially responsible investing, Ethical investing, ESG, CSR, Economic thought, Sustainable development, Environmental issues, European action plan on sustainable finance, Economic history

## Abstract

In this work, we present an overview of the historical development of socially responsible investing (SRI). We will argue that such a financial activity has been boosting in recent decades from a niche, mainly as a religious-led exclusionary practice, towards a mainstream strategy of risk analysis for institutional and retail investors. We also discuss the advances and possible drawbacks that regulatory activity and harmonization process on such industry have achieved at international level in recent years, with a special focus on the European Union. The study shows that the lack of a globally accepted taxonomy on what constitutes sustainable activities, of regulatory clarity and of high-quality data allowing for comparisons across industries and regions, together with practical and behavioural complexities are major critical issues that discourage SRI industry at the global level.

## Introduction

In this work, we present an overview of the historical development of socially responsible investing (SRI^1^) and evaluate the progress that regulatory activity and harmonization process concerning such financial industry have achieved at international level in recent years, with a special focus on the European Union.

According to the Global Sustainable Investment Alliance (GSIA), SRI is an investment approach that considers environmental, social and governance (ESG) factors in portfolio selection and management. GSIA uses an inclusive definition of SRI, without distinctions between this term and related ones, such as ethical investment, responsible investing and social investment. These are collectively referred to as *sustainable investing* or SRI.

As documented by the GSIA report of 2016, at the beginning of that year $22.89 trillion of assets were professionally managed under responsible investment strategies globally, representing 26% of all professionally managed assets, with an increase of 25% since 2014.

The reasons for this unprecedented growth path are at least twofold. First, the financial crisis of 2007–2008 has increased the awareness about the repercussions that weak corporate governance and risk management practices can have on financial markets and world’s economies. Second, the challenges entailed in the climate change process and depletion of natural resources (as well as air and water pollution and biodiversity loss) have increased demand for more responsible behaviour and coordination at global level from both public and private economic organizations.

As a response, several initiatives have taken place by international organizations to cope with such issues through global governance, which have provided a significant impulse to the SRI industry: for example, in 2006 the UN launched 6 Principles of Responsible Investment (UN PRI), and in 2015, it established 17 Sustainable Development Goals (SDGs), which set out ESG objectives for UN member states. At the Paris Climate Conference in 2015, 195 countries adopted a universal, legally binding global climate agreement, setting out a common action plan to avoid dangerous climate change, by limiting global warming to well below 2 °C and pursuing efforts to limit it to 1.5 °C.^2^ Finally, in several jurisdictions, pension funds, insurance companies and asset managers are required to report on whether and how they take ESG factors into account in their investment activities.^3^

On the other hand, growing empirical evidence has shown that ESG-related issues represent concrete sources of potential risk for investors, so that incorporating ESG criteria in investment strategies should become part of an overall risk analysis aimed at contributing to more stable financial returns.^4^ In the light of these findings, an increasing number of investors, both individual and institutional—such as pension funds, insurance companies and asset managers—have been paying heed to ESG factors in their investment strategies in the last two decades. Significantly, several private-sector organizations have been established to set out global principles for corporate disclosure and reporting on climate-related risks, although on a voluntary basis.

As a matter of fact, Dawkins (2016) underlines that “the important misgivings regarding the measurement of SRI notwithstanding, it is reasonable to conclude that there are very large sums of money in SRI funds and that it is a matter of importance to investors”.

However, a relevant obstacle to a widespread furthering of SRI is the lack of a universally agreed taxonomy of SRI practices and regulations. The latter elements, besides producing comprehensible confusion among individual investors and intermediaries, also raise concerns on so-called companies’ “green-washing” behaviour. Other open issues concerning SRI development are the lack of standardized data and metrics, given the presence of different classifications from sparse data providers,^5^ which prevents comparisons of SRI practices and performances across time and regions.

Since the early 1990s, different disciplines have been increasingly focusing on the analysis of SRI (see Rizzi et al., 2018 for recent literature), providing a deeper and wider understanding of such phenomenon. Although results are still far from being univocal from an economic point of view, SRI supporters claim that SRI not only allows investors to align their investment choices with their personal motivations, but also provides incentives to firms for voluntarily reducing their negative impact on societies.

While the dispute on SRI within the scientific community is still underway, the European Union is taking a primary role in relation to the global sustainability agenda.^6^ In particular, in recent years the European Commission has launched several ambitious proposals aimed at harmonizing and enhancing the ESG-factor integration within the European Union. These initiatives have produced an ongoing debate between private sector organizations and the European policymakers on what approach (either legislative or market-led, mandatory or voluntary), degree and type of disclosure is needed for institutional investors on ESG factors. For example, concerns have been raised that investment governance standards may be unduly restrictive and so discourage institutional investors from taking ESG factors into account in their analysis, even when ESG integration could lead to more resilient investment portfolios (OECD, 2017).

In this work, we will adopt a historical perspective for assessing the evolution of both the concept and practices of SRI, from the origins of mostly religious-related, exclusionary practices, towards a mainstream strategy of risk analysis for institutional investors. By doing so, we aim at shedding new light on the current debate concerning SRI and, in particular, on the above-mentioned initiatives that have been taken at European level, which may represent a significant step towards the furthering of the SRI industry.

The work is organized as follows: in Sect. 2, we introduce the concept of SRI and retrace the debate on ethical factors in the economic science, which will help understand the current debate within the scientific community and among practitioners on SRI. In Sect. 3, we will present and evaluate some recent packages proposed by the European Commission. Section 4 concludes the paper.

## The evolution of the concept of SRI

Currently, there is no generally agreed-upon definition on what SRI actually is. Indeed, many authors have documented that the definition of SRI is not univocal (Sparkes, 2001; Fowler & Hope, 2007; Chatterji et al., 2009; Busch et al., 2015), so that the issue of how to delimitate and quantify the phenomenon under investigation is worth being explored.

Nicholls (2010) proposes to consider SRI as a subset of the broader concept of “Social investment” or “Social finance” (SF). The latter, according to Rizzi et al. (2018), “defines the set of alternative lending and investment approaches for financing projects and ventures, requiring to generate both positive impacts on society, the environment, or sustainable development, along with financial returns […]. The term SF includes a variety of approaches, models and tools such as alternative currencies, community investment, crowd funding, ethical banking, microfinance, social impact bonds, social impact investing, socially responsible investment, venture philanthropy” (Rizzi et al., 2018, p. 805). SF, according to Nicholls (2010), encompasses three investment objectives: generating only social and environmental returns (for example, through government spending or support for social movements); generating pure financial returns to capital as in the case of conventional investing (for example, in clean energy stocks). “However, social investment is unique in including a third logic—blended value creation (see Emerson, 2003)—that combines both an attention to financial return and a focus on social/environmental outputs and outcomes. Blended value creation aims to challenge the Pareto assumption that achieving greater social/environmental impact inevitably reduces financial returns to capital (see Emerson, 2006 and Emerson & Spitzer, 2007). Examples of investment for blended value creation include socially responsible investment and mutual finance” (Nicholls, 2010, pp 75–76).

In the words of Puaschunder (2016), “financial social responsibility attributes the consideration of CSR [Corporate Social Responsibility, *N. o. A.*] in investment decisions […].^7^ Financial social responsibility bridges the financial world with society in socially responsible investment (SRI). In this asset allocation style, socially conscientious investors select securities not only for their expected yield and volatility, but foremost for social, environmental and institutional ethicality aspects” (p. 39).

Besides the objective difficulties to find a univocally accepted definition of SRI, at international level disparate SRI practices have emerged, influenced by specific national legislations, policy objectives and cultural customs (Reinhardt et al., 2008; Steurer, 2010), with the risk to make both the concept and the practices of SRI rather blurred and inconclusive and raising concerns for “green-washing” or “ESG-washing” as part of the barriers to SRI development. As a matter of fact, with no stringent legal international basis, SRI activities are in fact mandated by national, federal, state, or local laws and regulations.

Trying to clarify the issue concerning SRI practices, we recall that SRI encompasses activities that range from simple exclusions to general integration of ESG factors into traditional financial risk analysis. More precisely^8^:

- screening (negative/exclusionary screening, positive/best-in-class screening, norms-based screening);
- sustainability themed investing;
- impact investing;
- community investing;
- integration of ESG factors;
- corporate engagement and shareholder action/advocacy.

To some extent, the debate on the definition on SRI reflects the fact that, on one hand, both the scope and the practices encompassed by SRI have significantly changed over time and that, on the other hand, the ethical nature of SRI might be considered as too generic and muddled, dependent on the context (society) in which it is applied.^9^ Moreover, the economic science, especially in the last century, has been traditionally uncomfortable with “ethics”, for the reasons that we will discuss in the next sub-section.

### From ethical investment to socially responsible investment

Several authors emphasize the ethical foundations of SRI (in fact ethical investing was the term originally used to describe SRI—e.g. Domini, 1984; Simon et al., 1972) relative to conventional investing activity. For example, according to Richardson and Cragg (2010), SRI investors reflect the view that they have ethical responsibilities, so that they temper investment return by corporate responsibility, and to that end, they try to exert ethical influence over the firms they invest in. However, while “ethical investment” could in fact well describe the decision process of such value-based organizations in applying internal ethical principles to an investment strategy, some authors doubt that the same term could be applied to the profit maximizing behaviour of fund management companies supplying ethical unit trusts (Anderson, 1996). As Cowton (1994) posed it: “at one level, ethical investment can be seen as just another product innovation that helps widen choice […]. The irony is that its occurrence can be explained in pure, profit-seeking capitalistic terms, as financial institutions seek to influence and exploit their environment in the interests of profitability. Thus individual investors, potentially at least, have their values met or satisfied by institutions/people who do not share these values at all, whose sole motive might be to make more money” (p. 228).

Moreover, others consider the term “ethical” to be incorrect because it could imply that the mainstream approaches to investment are “unethical” (Purcell, 1980). Following this reasoning, the term “socially responsible investment” could allow to avoid such preconceptions and facilitate a broader, more positive approach to nonfinancial considerations being adopted by investors.

Revelli (2016) even argues that the recent evolution has led SRI towards a financial approach where ethics is guided by finance and proposes a model to incorporate the financial aspect of SRI in ethical values that should help formalize investments promoting impact measurement and extra-financial performance.

As mentioned, this debate finds its reason, among other things, in the difficulty of using the term “ethical” to describe investment issues or, more precisely, the difficulty of introducing ethical factors into economics and economic behaviour, a long-disputed issue that is worth being sketched in the next sub-section.

### The ethical factor in the economic science and finance

For a long time, the economic science has not been comfortable with ethical issues. The rich debate of nineteenth century within the economic science led to the formulation of new methodological and epistemological foundations, according to which economics is a real “science” that enjoys the same status of the experimental sciences.

According to this view, economics needed to be liberated from elements that are considered “outsiders” (ideological, ethical, metaphysical or political), in order to be able to identify and formulate—through the use of empirical observation, the logical-deductive method and the mathematical tool—economic laws that are general and invariant with respect to the types of organization of the economy and society.^10^ For example, Jevons (1888) wrote that he aimed to “treat Economy as a Calculus of Pleasure and Pain, the form which the science, as it seems to, must ultimately take" (*Preface*). By the same lines, Leon Walras, in his *Elements of Pure Economics*, claimed that “pure theory of economics is a science which resembles the physic-mathematical sciences in every respect” (1874 [2013], p. 71). According to this approach, individuals derive and seek their satisfaction from the relationship of pleasure or pain with things, while elements such as altruism, compassion and social connections disappear, although with some exceptions, from the description of social and economic behaviour. For example, Keynes (1904^3^) writes: “it’s true that our economic activities are subject to the influence of a variety of motives, which sometimes strengthen and sometimes counteract one another, [but] it is also true that in *economic affairs the desire for wealth exerts a more uniform and an indefinitely stronger influence* amongst men taken in the mass that any other immediate aim […]. It is legitimate and even indispensable to begin by tracing the result of this desire under the supposition that it operates without check” (p. 119).

Hence, the utilitarian methodology of economics could work without any reference to external moral values, as it shares with the classical Smithian vision (or the neoclassical interpretation of Smith’s view) the substantial coincidence between individual interest and collective interest (expressed through the famous metaphor of the “invisible hand”).^11^ Personal interest and pleasure are the result of the will and of personal freedom, but they are not mediated in any way by reference to other principles that inform human beings and their living together. Decisions and actions are also independent of any historical and institutional context.

The debate on the necessity to distinguish between “pure” and “applied economics” has continued in subsequent years, especially in the form of a debate between the proponents of *laissez faire* and those of state intervention; however, the principle of “neutrality” of economic science has not been basically questioned for quite a long time.

In fact, some authors have recently argued that this approach has been a reductionist interpretation of the classical economic thought. Amartya Sen (1987), for example, argues that “[o]ther parts of Smith's writings on economics and society, dealing with observations of misery, the need for sympathy and the role of ethical considerations in human behaviour, particularly the use of behaviour norms, have become relatively neglected as these considerations have themselves become unfashionable in economics” (p. 28).^12^

However, we can say that since the nineteenth century, the reductionist interpretation of the classical approach has prevailed, so that from that moment onwards economics would be completely separated from virtue and ethics. A famous example of such an approach is an article that Milton Friedman wrote for the *New York Times Magazine* in 1970,^13^ arguing that public companies possess only minimal ethical obligations (e.g. to operate within the societal ethical frame, to avoid deception and fraud) beyond maximizing profits and obeying the law.^14^ Friedman’s view is well in line with the traditional optimal portfolio theory pioneered by Markowitz (1952), according to which restricting an investment universe (for any reason) should be avoided in investment decisions because it would reduce the efficiency gains of diversification.

In support of Friedman’s view, Rudd (1981) argues that the performance of a constrained/screened portfolio, as in the case of SRI, is generally lower, in that, for example, SRI introduces size and other biases that, by reducing diversification, will produce relatively greater extra-market covariation in returns. Hence, Rudd questions the very legitimacy of social responsibility criteria: “there is one important difference between social responsibility criteria and others. The latter are imposed on the manager solely by the investment considerations. It is true that they may be misguided, but the underlying rationale is defensible; namely, the aim is to protect the financial condition of the beneficiaries. Few of the social responsibility criteria have this property” (p. 61). On the same lines, Grossman and Sharpe (1986) argue that any constraint placed on a portfolio can only reduce or leave unchanged the maximum utility obtainable through an investment decision.

However, in the last decades, several branches of the literature have tried to pursue alternative approaches to the mainstream neoclassical one, with emphasis on the psychological element. For example, behavioural economy unveiled, through experimental and fields studies, evidence of pro-social behaviour or limited rationality among individuals (Bénabou & Tirole, 2010; Meier, 2007). The role of institutions and of social capital (Keefer & Knack, 2008) and of CSR companies behaviour (Crifo & Forget, 2015) has been highlighted as crucially affecting individuals’ behaviour and collective outcomes, too.

To date, the research lines that have moved away from the so-called mainstream approach to use a less partial and more realistic view of the *homo oeconomicus*, while remaining within individualism and admitting different degrees of rationality or altruism, have not provided yet general models and theorems that can redefine fundamental economic phenomena such as production, consumption and distribution. Although lacking the descriptive and prescriptive power of the neoclassical theorems, these studies have opened promising avenues for research, which seem particularly suitable for modern economics and finance.

Finally, within the neoclassical framework, recent economic theory proves that social welfare might not be maximized if some externalities (e.g. environmental pollution) are not priced at all, so that public intervention is called for to correct such market-failure, typically through Pigouvian taxation or environmental regulation.^15^ In fact, a growing body of literature has shown that ESG factors can represent material risks for both productive companies and financial investors, so that their integration within the traditional risk analysis, while facilitating sustainable development, is increasingly considered essential for the achievement of successful performances in the long run.^16^

Also (or perhaps mainly) for all these reasons, the term “ethical investment” has increasingly been replaced, over the last ten years, by the term “socially responsible investment” or “sustainable investment”.^17^

#### History and evolution of SRI practices

Several authors have documented that SRI can be traced back to its religious origins, going back to the Hebrew Bible, over 2000 years ago.^18^

Social screening of investment opportunities has emerged in the religious communities of pre-revolutionary America, when Methodist communities, followed by the Quakers, screened out investment opportunities in the so-called “sin stocks”, that is companies involved in the industry of tobacco, gambling or alcohol, or were involved in slavery.

From the seventeenth until the mid-twentieth century, SRI remained a religiously centred, small movement and was basically focused on negative screening to avoid investment in “sin industries”.^19^

The protests of students and young people against the Vietnam War in the 1960s led to a boycott campaign against companies that provided weapons used in the war and alerted institutional investors to sell napalm-producing Dow Chemical shares (Biller, 2007).^20^ The latter campaigns, together with protests and proposals on civil rights and democratic participation, helped SRI to come out from the universe of religious activism, so that, together with the establishment of Community development banks that were settled in low-income or minority communities, they started to be identified as the beginning of modern SRI.^21^

In 1971, the *First Spectrum Fund* was established, promising no investment would be made without analysing companies’ performance in “the environment, civil rights and the protection of consumers”. On the same lines, the *Dreyfus Third Century Fund*, established in 1972, had a prospectus stating that it was looking for companies that “show evidence in the conduct of their business, relative to other companies in the same industry or industries, of contributing to the enhancement of quality of life in America”. In the mid-1970s, Reverend Leon Sullivan, a civil rights activist and director of *General Motors*, organized a broad movement of opinion and organization of the shareholding of some large corporations and launched investment principles whereby US companies operating in South Africa would have to apply to their local workers the same rules as those for American employees. The investment principles launched by Sullivan were followed by massive financial boycott and extensive pressure on the managers and boards of American multinationals involved in South Africa.

In 1982, Joan Bavaria founded the *Trillium Asset Management*, a firm that defines itself as the “oldest independent investment advisor devoted exclusively to sustainable and responsible investing”.

In the following years, SRI was focused on screening initiatives in South Africa with the goal of pressuring the South African government to end *apartheid*, as well as on divestiture strategies against the Angolan repressive government.^22^ In addition to playing a role in political activism, in the same years SRI began to increase its influence in mainstream investment, with positive screening strategies starting in the beginning of the 1990s.

One of the first SRI indexes is *The Domini 400 Social Index* (now MSCI KLD 400 Social Index) launched in May 1990, a capitalization weighted index of 400 US securities that provides exposure to companies with outstanding ESG ratings and excludes companies whose products have negative social or environmental impacts. Among the other currently existing indexes, we want to cite the *Dow Jones Sustainability Index*, *Ethibel*, *FTSE4*, *Humanix* and *Jantzi*.

In 2015, the US Department of Labor issued a clarifying guidance on ESG integration, confirming that fiduciaries may legitimately consider ESG factors if they have an impact on financial risks and provided that the overall decision-making process is in line with existing standards.

Similarly, as for non-US countries, the 2011 Amendment to the Pension Funds Act of South Africa states that “prudent investing should give appropriate consideration to any factor which may materially affect the sustainable long-term performance of a fund’s assets, including factors of an environmental, social and governance character”.

As for the European market, UK has the largest number of SRI funds in Europe, although the first ethical retail fund in Europe (i.e. available to all investors) was *Ansvar Aktiefond Sverige*, founded in 1965 in Sweden (Mill, 2006).

The above documented development of SRI, while providing new opportunities in terms of financial social market options, also caused a large disparity of SRI practices. For these reasons, in early 2000s the United Nations have launched a program using a bottom-up (or more pragmatic) approach, inviting a group of the world’s largest institutional investors to join a network aimed to codify SRI practices and put them into practice. Signatory institutions were asked to subscribe six *Principles of Responsible Investments* (PRI), launched in April 2006 at the *New York Stock Exchange* and concerned with ESG issues, such as human rights and climate change, that are meant to provide a general framework for mainstream investors to consider these ESG issues. Initiatives for boosting SRI at global level have also been taken by the World Bank and the International Finance Corporation, which are major issuers of labelled and themed bonds through their *Sustainable Development Bonds* and other structured products.

However, in most OECD countries regulatory frameworks for institutional investors (pension funds, insurance companies and asset managers) do not make explicit reference to ESG factors, although with some notable exception. In general, incorporation of ESG factors in investment governance is allowed to the extent that they are expected to have a material impact on financial performance. Consequently, it is up to institutional investors to decide whether and to what extent ESG integration is consistent with the usual standards of behaviour, such as prudence and risk control.

A relevant exception is presented by the French legislation, according to which from 2017 institutional investors (and—more in general—companies), must report on the financial risks they face in relation to the consequences of climate change and on the measures taken to reduce these risks.

As for corporate disclosure on ESG factors, in several countries companies are required to report on whether and how they take ESG factors into account, although in most cases it is not mandatory (i.e. in the form “comply or explain”).^23^ On the other hand, a number of organizations and initiatives (also practitioner-led ones) have emerged in recent years in order to facilitate the consistent disclosure and integration of ESG factors by companies and asset managers. For example, the Financial Stability Board’s (FSB) *Task Force on Climate-related Financial Disclosure* (TCFD) initiative has the objective since 2015 to deliver voluntary, consistent climate-related financial risk disclosures to be used by companies in providing information to investors, lenders, insurers and other stakeholders. The final report of the TCFD was released in June 2017, containing four widely adoptable recommendations on climate-related financial disclosures that are applicable to organizations across sectors and jurisdictions.

Another recent official initiative is the Central Banks and Supervisors *Network for Greening the Financial System* (NGFS), launched, on a voluntary basis, at the Paris *One Planet Summit* in December 2017.

As a comment, it is worth noting that although a major critique against the above-mentioned initiatives is their voluntary nature and their lack of external monitoring and sanction mechanisms, some studies have shown that investors may be able to pressure signatories to increase their compliance with the disclosure requirements and to “walk the talk”.^24^

However, regulatory, practical and behavioural barriers to more widespread SRI remain. The lack of harmonization and common definitions is one of the most relevant obstacles, because interpretations of ESG vary within the investment communities and across jurisdictions. Moreover, many asset owners argue that the narrow interpretation of fiduciary duty, which considers ESG factors to be nonfinancial and therefore in conflict with the duties of care and loyalty, is still influential, especially in common law countries and, hence, is a primary obstacle to ESG integration (PRI, 2015).

## SRI in the European Union

As highlighted in Eurosif (2018), in the European Union the increase in demand for SRI today, also from retail clients, is not matched by adequate product offer, given the difficulties arising from specific legislation concerning financial services, such as the Markets in Financial Instruments Directives (MIFID I and II) and the Regulation on Markets in Financial Instruments (MIFIR). In fact, the latter acts still contain no specific requirements to embed ESG factors as part of the investment preferences discussed with the client. Moreover, there are also behavioural motivations due to the fact that many financial advisers still today perceive SRI as presenting a negative trade-off with returns — although the latter is not necessarily true (see Renneboog et al., 2008 for insightful reviews).

For these reasons, in recent years the European Union has undertaken several steps to increase the availability of green funds and boost SRI, which we summarize in the next subsection.

### The European framework for sustainable development

In recent years, the European Commission has launched several initiatives on sustainable development within a framework aimed at putting ESG factors at the heart of the financial system. The aim is to help transforming Europe's economy into a greener, more resilient and circular system.

The framework has its roots in the 2015 Paris agreement on climate change (COP21), the United Nations (UN) 2030 Agenda for Sustainable Development and the EU 2030 Framework for climate and energy, adopted in October 2014. This framework includes also the Investment Plan for Europe, the Circular Economy Package, the Energy Union package, the Capital Markets Union and the EU budget for 2014–2020, comprising the Cohesion fund and research projects.^25^

To sum up, in September 2016, the European Commission proposed the extension of the duration of the European Fund for Strategic Investment (EFSI) until 2020, and in November 2017 the Council confirmed the agreement on such extension (referred to as EFSI 2.0), by earmarking at least 20% of the EU 2014–2020 budget available for climate action. The agreement increases the EU guarantee from 16 billion euro to 26 billion euro and the European Investment Bank (EIB) capital from 5 to 7.5 billion euro, with the objective of mobilizing 500 billion euro of both private and public investment.

Second, in late 2016 the European Commission established the EU *High-Level Group on Sustainable Finance* to help develop an overarching and comprehensive EU roadmap on sustainable finance. The Group completed its work by publishing a final report in January 2018, suggesting eight recommendations—including the establishment of a EU sustainability taxonomy, i.e. a technically robust classification system at EU level to provide clarity on what is “green” or “sustainable” activity. These recommendations have been at the heart of the initiatives that the European Commission has launched in the subsequent months:

- in March 2018, the publication of the *Action Plan on Sustainable Finance*, with the objective of reorienting capital flows towards sustainable investments in order to achieve sustainable and inclusive growth;
- in May 2018, a package of measures implementing several key actions contained in the action plan.

The latter package is meant to integrate sustainability into the suitability obligations arising from several EU Directives, among which the 2014/65/EU (MiFID II) on financial services and the EU Directive 2016/97 (IDD) on insurance distribution. In particular, it includes a proposal for a regulation that establishes the conditions and the framework to gradually create a unified classification system (“taxonomy”) on what can be considered an environmentally sustainable economic activity, to create EU labels for green financial products, to clarify fiduciary duties of asset managers and institutional investors, by introducing disclosure obligations on how institutional investors and asset managers integrate ESG factors in their risk processes, to enhance corporate reporting on climate issues. Finally, in order to help implement its action plan, the Commission set up a *Technical Expert Group on Sustainable Finance* (TEG) with the specific target of developing:

- a EU classification (taxonomy) on environmentally sustainable activities (to be finalized by the end of 2022 by the Commission);
- a EU Green Bond Standard;
- benchmarks for low-carbon investment strategies;
- guidance to improve corporate disclosure of climate-related information.^26^

In September 2017, the European Commission presented a series of proposals to improve the mandates, governance and funding of the European Supervisory Authorities (ESAs) for banking (European Banking Authority, EBA), for securities and financial markets (European Securities and Markets Authority, ESMA), and for insurance and pensions (European Insurance and Occupational Pensions Authority, EIOPA). In particular, the proposal specifically requires the ESAs to take into account ESG factors arising within the framework of their mandate, thus promoting sustainable finance while ensuring financial stability. This proposal is meant to enable the ESAs to monitor how financial institutions identify, report and address risks that ESG factors may pose to financial stability. Moreover, according to such proposals the ESAs will also provide guidance on how EU financial legislation can integrate sustainability considerations and promote the implementation of these rules.

To conclude it is worth remembering that in January 2019, the European Commission published draft rules on how investment firms and insurance distributors should take sustainability issues into account when providing their services. Although the Commission can only officially adopt these draft rules once the proposed package has been approved at EU level, the draft rules are meant to help investment firms and insurance distributors to already prepare to take ESG considerations and preferences into account in their investment advice and portfolio management, and into the distribution of insurance-based investment products.

### The current debate on the EU regulatory initiatives

While public consultations on the proposed packages are still underway, concerns have been raised by category organizations such as the *European Fund and Asset Manager Association* (EFAMA, 2016). The latter, while supporting the goal of enhancing ESG factors disclosure and the proposal of a EU taxonomy, recommends flexibility to allow for innovation and client-driven developments. EFAMA considers SRI as a young, innovative and still developing field, which cannot be captured by a single regime. Hence, it argues that a prescriptive legislative approach, unlike a market-led or self-regulatory approach, will create unintended barriers to market development. Moreover, it claims that the ESG reporting should be an activity of the asset-owner, rather than of the fiduciary investor. The same advice to avoid inflexible or overly prescriptive regulations has been raised by the *Securities and Markets Stakeholder Group* (SMSG).

As for the proposed amendment of ESAs mandates, some NGO organizations, such as *Finance Watch* and *ShareAction*, expressed a negative judgment, arguing that the final legislation should detail a clear mandate—tasks and powers—for the ESAs to conduct analysis on a European-wide basis of ESG factors and ESG considerations into their roles. Also, the European Parliament has criticized the Commission’s proposal, since allegedly the proposal’s chosen approach to ESAs reform is not in line with the impact assessment that the Commission conducted on the topic earlier.

As for the IORP II Directive, it is worth noting that while it includes several new ESG provisions related to areas such as risk management, it does not require the integration of ESG criteria in investment decisions.

However, on 24 July 2018 the European Commission has formally asked EIOPA and ESMA to deliver technical advice (comprising a cost–benefit analysis) on two delegated acts aimed to incorporating sustainability risks (i.e. ESG risks) in the decisions taken and processes applied by financial market participants subject to those rules. On 3rd May 2019, EIOPA submitted its advice to European Commission stating that “sustainability is an area of significant strategic importance. Consequently, EIOPA strongly supports the European Commission's Sustainable Finance Action Plan including the aim to integrate sustainability considerations into the prudential and conduct framework for insurers, reinsurers and insurance distributors”.^27^

Some member states and associations (such as *PensionsEurope*, representing national associations of pension funds and similar institutions for workplace pensions) have asked that the delegated act concept be deleted from the IORP II Directive amendment proposal, their concern being that amendments made via delegated acts would result in prescriptive rules without any scope for national implementation. Moreover, they oppose the possibility that through delegated acts the same set of rules would be issued for both insurance companies and pension funds. Moreover, they argued against changes to IORP II as the directive—at that time—was still under implementation.

In November 2018, the European Parliament’s economic and monetary affairs committee decided to allow delegated acts under IORP II amendment, while in December 2018, the EU Council has dropped the disputed amendment to the IORP II Directive allowing for delegated acts. Moreover, the texts from the Council and Parliament have differing definitions of sustainability risks. According to *PensionsEurope*, the latter’s definition even aims to mandate pension schemes to account for externalities in investment decisions.

The Council’s agreement on the disclosure proposal (and on the Commission’s low carbon benchmarks proposal) implies, in any case, that negotiations between the Parliament and the Council, with the Commission acting as arbiter, can begin. In fact, the final sustainable finance legislation will depend on such negotiations, with the Council Presidency and the Parliament to decide the date of start.

As a final comment, while we think that some concerns emerged from institutional investors are sound and need careful consideration, we believe that the initiatives undertaken by the European Union are going in the right direction. On the one hand, the challenges concerning social and environmental sustainability urgently require rapid and decisive interventions for the improvement of the well-being of European society; on the other hand, the success of these regulatory proposals, although capable of possible improvements, may represent a benchmark for the other jurisdictions and for future generations.

## Conclusions

Despite the structural difficulties of the economic science in taking ethical factors properly into account, the socially responsible investment industry has been flourishing in the last decades, based, on one hand, on the increased demand from retail investors for more responsible behaviour of financial and business sectors and, on the other hand, on the evidence that ESG factors are a material risk for financial investors. All these elements have induced an increasing number of firms to incorporate ESG factors within their overall risk analysis to get more stable financial returns. However, more work on several issues is needed to help a full development of SRI. The lack of a globally accepted taxonomy on what constitutes sustainable activities and of regulatory clarity, practical complexity and behavioural issues are all critical aspects that discourage ESG integration. These elements need to be coupled with increased transparency,^28^ defined standards and improvement of high-quality data availability across industries and regions. In this respect, the establishment of an accountability or overarching governing body could ensure accuracy of reported information and favouring a coordination of the existing corporate reporting initiatives promoted by international associations towards common frameworks. Finally, we have discussed several proposals that the European Union has launched in recent years, within a long-term sustainability framework explicitly following the path paved by the 2015 Paris agreement on climate change and the UN 2030 Agenda for Sustainable Development. The EU initiatives are particularly relevant, although far from being fully implemented: the debate about the trade-off between harmonization and regulatory requirements, on one hand, and freedom and flexibility needs expressed by the financial and insurance associations, on the other hand, is rich and alive.

The way in which the EU will tackle such issues in the next years is likely to represent a significant turning point for the enhancement of SRI at global level and the improvement of well-being for future generations.

## Footnotes

1 For the sake of brevity, the abbreviation SRI in this text stands for both “Socially Responsible Investing” and “Socially Responsible Investment”.

2 The EU has estimated an investment gap of 180 billion euro per year in order to achieve those targets.

3 Updated lists of country-level initiatives and international organizations are contained in Blackrock (2016) and OECD (2017), and, as for EU countries, in Eurosif (2018) and Kahlenborn et al. (2017).

4 The empirical link between environmental degradation, economic growth and financial development has been extensively analyzed in recent years. For example, economic growth, trade openness and foreign direct investment are generally found to increase environmental degradation (Nasir et al. 2019; Pham et al. 2020), while financial development and energy use tend to increase CO_2_ emissions. On the other hand, R&D expenditures and energy research innovations reduce them (Shahbaz et al., 2019, 2020; Nguyen et al. 2021). Nasir et al. (2021) find that short-term bidirectional causality prevails between economic growth, energy consumption, industrialization and stock market development with CO_2_ emissions. Doğan et al. (2020) find that renewable energy is more suitable for economic growth than non-renewable.

5 OECD (2017).

6 See Parker and Karlsson (2017).

7 According to Sparkes (2002), “corporate social responsibility (CSR) and socially responsible investing are in essence mirror images of each other. Each concept basically asserts that business should generate wealth for society but within certain social and environmental frameworks. CSR looks at this from the viewpoint of companies, SRI from the viewpoint of investors in those companies” (p. 42). See also Marinetto (1999) and Stutz (2018).

8 See Inderst and Stewart (2018) for a deeper insight.

9 For a deeper analysis of the reasons behind the heterogeneity both in the definitions and practices of SRI, see Sandberg et al. (2009).

10 Sachs (2013), p. 86 and Guidi (2004).

11 On this point see Martini and Spataro (2018).

12 See, for example, Smith (1853).

13 The title of the article is self-explaining: “The Social Responsibility of Business Is to Increase Its Profits”.

14 Hill et al. (2007).

15 See, for example, Renström et al. (2020, 2021) and Wang et al. (2020).

16 See, for example, Laurel-Fois (2018).

17 It is worth mentioning that some authors argue that the mainstreaming of SRI has transformed the original goal of “making good” into a quest for profitability (Hellsten and Mallin, 2006) and call for a return of SRI back to the primary virtuous logic of a “margin” or niche market (Revelli, 2017).

18 See, for example, Ciocchetti (2007), pp. 1976–1977. During the centuries, Judaist writings praised ethical monetary conduct and usury was prohibited by the Catholic Church in 1139. Until today Islamic banking has restricted adult entertainment and gambling (Renneboog et al., 2008).

19 The first public offering of a screened investment fund was in 1928 when an ecclesiastical group in Boston established the *Pioneer Fund*.

20 See the *PAX World Fund*, founded in 1971 by the Methodist clergy, that was aimed at divestiture from Vietnam War supporters (Broadhurst et al., 2003; Renneboog et al., 2008).

21 Solomon et al. (2002); Sparkes and Cowton (2004).

22 See Schueth (2003).

23 OECD (2017).

24 See, for example, for the UN Global Compact, the analysis carried out by Amer (2018).

25 See the website of the European Commission for further reference: https://ec.europa.eu/info/index_en.

26 The TEG operated until 30 September 2020 and after the beginning of the Covid-19 crisis has issued a specific statement and a short paper (“5 high-level principles for Recovery & Resilience”), encouraging governments and the private sector to use EU Taxonomy, the Green Bond Standard and the Climate Benchmarks as tools to ensure a 'resilient, sustainable and fair' recovery (see https://ec.europa.eu/info/publications/sustainable-finance-technical-expert-group_en).

27 https://www.eiopa.europa.eu/content/eiopa-submits-advice-sustainable-finance-european-commission_en.

28 See Gupta and Mason (2014), Alcaraz-Quiles et al. (2019).
